# Evaluating a Novel Method to Limit Non-Target Mortality in Attractive Toxic Sugar Bait Systems

**DOI:** 10.3390/insects17040370

**Published:** 2026-04-01

**Authors:** Dongmin Kim, Liam C. Shine, Tanise Moitinho S. Stenn, Bryna C. Wilson, Eric P. Caragata, Barry W. Alto, Nathan D. Burkett-Cadena

**Affiliations:** Florida Medical Entomology Laboratory, University of Florida, Vero Beach, FL 32962, USA; kimdongmin@ufl.edu (D.K.); lshine@ufl.edu (L.C.S.); tanise@ufl.edu (T.M.S.S.); brynawilson@ufl.edu (B.C.W.); e.caragata@ufl.edu (E.P.C.); bwalto@ufl.edu (B.W.A.)

**Keywords:** attractive toxic sugar baits, perforated bag, mosquito sugar feeding, mosquito control, non-target effects, propylene glycol

## Abstract

Attractive toxic sugar baits (ATSB) exploit mosquito sugar-feeding as a control strategy, but their use has been hindered by non-target (pollinator) exposure, environmental degradation, and the difficulties of selectively administering toxicants to mosquitoes. We developed and evaluated a perforated bag-based ATSB system incorporating 200–300 microperforations (<0.25 mm in diameter) to selectively deliver toxicants in sugar water to mosquitoes while physically excluding non-target pollinators (i.e., butterflies and honey bees). In laboratory assays, the perforated bag design supported high mosquito feeding success (87%) and rapid mortality (100% within 24 h) when combined with propylene glycol or dinotefuran, without deterring feeding behavior. Butterflies and honey bees were unable to access sugar solutions through the perforated bag, demonstrating selectivity toward mosquitoes due to differences in mouthpart morphology compared to non-targets. Semi-field trials showed that integrating perforated ATSB system into passive traps did not reduce capture rates of gravid *Culex quinquefasciatus* or host-seeking *Aedes aegypti*, while inducing near complete mosquito mortality (>90%) following capture. Our results indicate that perforated bag-based ATSB systems incorporated into passive traps can target mosquitoes across multiple physiological states while minimizing the non-target risk. This platform provides promising and environmentally responsible integration of ATSB deployment into mosquito surveillance and control programs.

## Simple Summary

Mosquitoes spread pathogens that affect millions of people worldwide. One way to control mosquitoes is by exploiting their natural sugar-feeding behavior. Attractive toxic sugar baits (ATSB) use a sugar solution containing a toxin to attract and kill mosquitoes, but these baits can also harm beneficial insect pollinators such as butterflies and bees. In this study, we evaluated a new ATSB design that uses a thin membrane with very small holes. These holes allow mosquitoes to feed while preventing larger insects, lacking piercing-sucking mouthparts, from accessing the sugar. We assessed mosquito feeding success, mosquito mortality, and the protection of butterflies and honey bees. We found that mosquitoes readily fed on the ATSB and experienced high mortality, whereas butterflies and honey bees were unable to access the ATSB. Our results demonstrate that simple physical barriers can reduce non-target exposure while maintaining effective mosquito control. This approach may contribute to safer mosquito management strategies that better protect insect pollinators and the environment.

## 1. Introduction

Mosquitoes transmit numerous arboviruses that cause illnesses ranging from mild symptoms to death [1,2]. In the United States, state and local agencies protect public health through year-round mosquito surveillance and control, using an integrated mosquito management (IMM) approach. Adulticide applications, such as ultra-low volume (ULV) spraying, remain a primary tool for rapidly reducing adult mosquito populations and preventing arbovirus transmission. However, public concerns about potential impacts of adulticides on non-target organisms (e.g., butterflies, bees, and other beneficial insects) often influence mosquito control decisions. These concerns, combined with increasing insecticide resistance amongst local mosquito populations, the logistical challenges (e.g., labor and cost) of repeated spray applications, and the robust rebound effect of mosquito populations [3,4], highlight the need for more sustainable and targeted control methods.

One promising mosquito control strategy is the use of attractive toxic sugar baits (ATSB) to attract and kill adult mosquitoes by exploiting their innate sugar feeding behavior [5]. The concept was first demonstrated by Lea [6], who showed that filter papers impregnated with malathion in 20% sugar (sucrose) or syrup solution caused significantly higher mosquito mortality (85.2%) than insecticide alone. Sugar feeding in mosquitoes provides the energy required for endogenous physiological processes and behaviors (e.g., flight, host-seeking, mating, blood feeding) [7] and nearly all species obtain these sugars from plant-derived sources, particularly nectar [8,9], honeydew [10], fruits [11], and plant tissue [12]. An ATSB delivers insecticides orally by combining a sugar solution with active ingredients such as deltamethrin [13], eugenol [14], boric acid [15], or spinosad [16]. For example, laboratory bioassays demonstrated that guava juice-based ATSBs containing deltamethrin achieved up to 98% mortality in *Anopheles stephensi* within 24 h, supporting their potential for mosquito control [13].

Building on this principle, field studies conducted across diverse ecological settings, including Israel, coastal Florida (USA), and multiple sites in Africa, have demonstrated that ATSB applications can reduce local mosquito densities by 50–90%, with residual efficacy lasting up to four weeks. Reductions have been documented across several medically important genera, including *Anopheles*, *Aedes*, and *Culex* [17,18,19]. For example, in Mali, ATSB interventions reduced *Anopheles gambiae* sensu lato populations by 90%, impacting the density and longevity of epidemiologically important older females [20]. The previous study shows how lethal and sublethal effects of ingestion of ATSB can yield favorable outcomes for mosquito control and public health via population suppression and life-shortening effects. That is, both ATSB-induced lethality and ATSB-induced life-shortening effects (i.e., ingestion of sublethal dose) [16,21] can cause a shift in the age-structure of the population to reduce older mosquitoes and mitigate transmission of pathogens. Compared to conventional adulticides, ATSBs offer specific advantages: (1) they target both sexes, (2) they may help mitigate insecticide resistance through rotation of active ingredients, and (3) they can be deployed passively in bait stations.

Conventional ATSB delivery methods, such as spraying toxic sugar bait onto vegetation, raise concerns about unintended impacts on non-target nectar feeders, which are common amongst diverse insect taxa and include ecologically important pollinators such as butterflies and bees [22]. Although a few studies have reported measurable effects of ATSB on non-target insects [14,23,24], these investigations were limited in the application methods evaluated, geographic scope, taxa surveyed, and duration of follow-up surveillance. Furthermore, the potential for cascading ecological effects remains poorly understood; for example, no studies have assessed whether predatory arthropods experience lethal or sublethal effects after consuming prey that had ingested ATSB toxicants. Additionally, environmental factors impose significant constraints on ATSB performance. Rain and dew can wash bait residues from vegetation, while ultraviolet (UV) radiation from sunlight can photodegrade both the sugar matrix and active ingredients [25]. High ambient temperatures may accelerate volatilization or chemical breakdown, and wind can physically displace droplets from treated surfaces. Collectively, these abiotic factors reduce bait persistence, decrease the likelihood of mosquito contact, and shorten the effective control period under field conditions.

To address these specific limitations of ATSB usage in mosquito control, we developed and evaluated a perforated bag-based ATSB system, implemented using a perforated bag design, to minimize unintended impacts on non-target arthropods while selectively targeting vector mosquitoes (Figure S1A). The specific objectives of this study were to: (1) quantify mosquito feeding success across different membrane types; (2) evaluate multiple active ingredients to optimize mosquito feeding and mortality; (3) assess non-target impacts on representative pollinators (i.e., butterflies and honey bees); and (4) test the perforated bag-based ATSB system under semi-field conditions targeting both gravid and host-seeking mosquitoes. By enclosing the toxic sugar bait within a protective membrane, a major goal of the platform is to facilitate more targeted and efficient control of adult mosquitoes while minimizing environmental impact.

## 2. Materials and Methods

### 2.1. Maintenance of Mosquitoes

Laboratory colonies of *Culex quinquefasciatus* Say (Vero Beach–Gainesville strain, 2015) and *Aedes aegypti* L. (Orlando strain, 1952) were maintained in an environmental chamber at the Florida Medical Entomology Laboratory (FMEL), University of Florida, Vero Beach, FL, USA, under controlled conditions of 27.0 ± 0.5 °C, 70.0 ± 5.0% relative humidity, and a 14:10 h (light:dark) photoperiod. Mosquito larvae (~150) were reared in plastic trays (24.8 × 19.7 × 3.8 cm) containing 1.0 L of tap water and fed a 1:1 mixture of brewer’s yeast and lactalbumin following a standardized rearing protocol [26]. Pupae were collected daily, placed into 30 mL emergence cups (50 pupae per cup), and transferred to mesh-screen cages (BugDorm; MegaView Science Co., Ltd., Taichung, Taiwan) for adult emergence.

### 2.2. Mosquito Sugar Feeding Across Membrane Types, Sugar Concentrations, and Thermal Cues

We first evaluated several different types of membranes to identify those eliciting the highest sugar-feeding success in mosquitoes. Tested membranes included cell culture bags composed of fluorinated ethylene propylene (FEP; OriGen, Austin, TX, USA), dialysis film (EaseToU, San Diego, CA, USA), DuraSeal film (Diversified Biotech, Dedham, MA, USA), Parafilm (Bemis Company, Inc., Neenah, WI, USA), Parafilm supplemented with adenosine triphosphate (ATP), polyisoprene (Suretex, Bangkok, Thailand), polyethylene film (Houseables, Reno, NV, USA), lambskin (Trojan, Trenton, NJ, USA), and latex (Trojan, Trenton, NJ, USA). Two delivery methods were used: flat films (e.g., DuraSeal and dialysis film) were stretched over 50 mL Falcon tubes and inverted into modified paper cup holders, while elastic and biological materials (latex and lamb skin) were filled with sugar solution and tied into sachets. Each membrane enclosed a 10% (*w*/*v*) sugar solution (sucrose) dyed with blue food coloring (50 µL per 10 mL sugar solution; McCormick & Company, Inc., Hunt Valley, MD, USA), which served as a visual indicator of sugar feeding. In a no-choice assay, 20 female *Cx. quinquefasciatus* (3–7 days old, starved of sugar and water for 24 h) were released into 32.5 × 32.5 × 32.5 cm mesh cages inside an environmental chamber and given unrestricted access to a single membrane type for 24 h. A sucrose-saturated cotton pledget containing the same 10% (*w*/*v*) sucrose solution dyed with blue food coloring served as the control. Following exposure, mosquitoes were immobilized by cold anesthesia (–20 °C), and feeding success was assessed by direct visual inspection of intact abdomens under a dissection microscope for the presence of blue food coloring.

The perforated bag-based ATSB system (Figure S1A) was adapted from Kim et al. [27] with modifications for sugar-feeding assays. Briefly, a 4.0 × 4.0 × 0.5 cm sponge (Amazon, Seattle, WA, USA) was inserted into a 5.08 × 7.62 cm resealable clear plastic bag (Amazon, WA, USA). One side of the bag was perforated by rolling a 0.25 mm microneedle roller (Sdara Skincare, Los Angeles, CA, USA) back and forth to create multiple (200–300) small holes. The bag was then filled with 20 mL of either 10% or 40% sucrose solution containing blue food coloring. After filling, the bag was gently compressed to expel air, ensuring the sucrose solution saturated the perforations. In preliminary experimentation we observed that sugar water did not leak through the 0.25 mm holes, while tap water leaked through. Hand warmers (Kobayashi Americas, Duluth, GA, USA) were positioned above the perforated bag for each 10% and 40% sucrose treatment, with and without thermal stimulation, to simulate vertebrate body temperature and evaluate the effect of thermal cues on sugar feeding. Controls included a sucrose-saturated cotton pledget (positive) and a non-perforated bag containing sucrose solution (negative). Feeding assays were conducted as described above to quantify mosquito feeding success (Figure S1B).

### 2.3. Acute Toxicity of ATSB Active Ingredients

We evaluated the feeding success and mortality of *Cx. quinquefasciatus* mosquitoes exposed to various commercially available active ingredients delivered via perforated bags. The tested compounds included boric acid (99.6%; Thermo Fisher Scientific, Waltham, MA, USA), dinotefuran (97.5%; Thermo Fisher Scientific, MA, USA), spinosad (0.5% spinosyn A and D; Southern AG Insecticides, Inc., Palmetto, FL, USA), and propylene glycol (99.9%; Glycerin Supplier, Houston, TX, USA). Boric acid was obtained in powder form, while spinosad, dinotefuran, and propylene glycol were liquid formulations. Each compound was prepared in a 10% sucrose solution supplemented with 0.1 mL of blue food coloring [28]. Final treatment concentrations were 5% boric acid, 1 mg/mL dinotefuran, 1 mg/mL spinosad, and 10% propylene glycol. For each treatment, twenty 3–7-day-old female *Cx. quinquefasciatus*, starved for 24 h, were released into 32.5 × 32.5 × 32.5 cm mesh cages (three replicates per treatment) maintained in an environmental chamber (27.0 ± 0.5 °C, 80.0 ± 5.0% relative humidity). Mosquitoes were given continuous access to the treated sucrose solutions via perforated bags for 72 h. Mortality was recorded every 24 h. Control groups were provided with sucrose-saturated cotton pledgets containing sucrose only and no active ingredients, serving as a conventional laboratory sugar-feeding control.

### 2.4. Sugar Feeding Success and Mortality of Insect Pollinators on Perforated Bag-Based ATSB System

To assess the accessibility of sugar solutions in perforated bags to non-target wild butterflies, five species (*Danaus plexippus*, *Danaus gilippus*, *Anartia jatrophae*, *Heliconius charithonia*, and *Ascia monuste*) were captured in Indian River County, Florida. In a no-choice assay, seven butterflies (mixed species) and 20 female *Cx. quinquefasciatus* (3 to 7 days old, starved for 24 h) as a control group were released together into 32.5 × 32.5 × 32.5 cm mesh-screen cages housed in an environmental chamber (five replicates). Both mosquitoes and butterflies were given unrestricted access to perforated bags containing a 10% sucrose solution only (no active ingredients), dyed with blue food coloring, for 48 h to assess feeding success. A sucrose-saturated cotton pledget was used as a control to confirm feeding access. Following exposure, butterflies were immobilized using cold anesthesia at −20 °C, and feeding success was assessed by gently compressing the abdomen with a serological pipette. The presence of blue dye in the abdomen was used as an indicator of feeding, evaluated under a dissection microscope (Figure S1C).

To evaluate the accessibility and lethality of a 10% sucrose solution containing 10% propylene glycol to honey bees (*Apis mellifera*; sourced from The BeeRing, Vero Beach, FL, USA), a no-choice assay was conducted using perforated bags. Groups of ten honey bees were placed in mesh-screen cages (approximately 150 µm mesh) and exposed to either perforated or non-perforated bags containing the sucrose–propylene glycol solution for 72 h. A cotton pledget saturated with the same sucrose solution without propylene glycol served as a control. The same experimental setup was applied to *Cx. quinquefasciatus* mosquitoes for comparison. Feeding success in honey bees was assessed by gently compressing the abdomen and inspecting for the presence of ingested solution (blue coloration) under a dissection microscope (Figure S1D).

### 2.5. Semi-Field Validation of Perforated Bag-Based ATSB System

The perforated bag-based ATSB system was evaluated under semi-field conditions using Biogents GAT (BG-GAT) traps (Biogents, Regensburg, Germany), which are passive traps designed to capture mosquitoes (Figure S2). Trials were conducted at the Florida Medical Entomology Laboratory (FMEL), Vero Beach, FL, USA. Each trap was fitted with a perforated bag containing a 10% sucrose solution and 10% propylene glycol, placed inside the barrier net to serve as the active ATSB formulation. Traps were baited with either a 72-h fermented grass infusion to attract gravid *Cx. quinquefasciatus* or an ExHale CO_2_ bag (20.32 cm × 12.7 cm × 35.56 cm, 1.81 kg; pre-activated version; Garden City Fungi, Missoula, MT, USA) to attract host-seeking *Ae. aegypti* [29]. For *Cx. quinquefasciatus* trials, three-day-old females were fed on a live chicken (IACUC protocol 201807682), after which fully engorged females were collected, maintained in mesh cages for 72–96 h, and subsequently used in the experiments. Control traps contained perforated bags without the active ingredient. Each trial consisted of 20 mosquitoes, with one treatment trap and one paired control trap, deployed in separate 203.2 × 203.2 × 157.5 cm screen enclosures (Folding Mosquito Net; Jsanh, Yangjiang, China) within the semi-field facility for a 24-h period (1500 to 1500 h). After exposure, mosquitoes captured in each trap were transferred to an environmental chamber and held for an additional 24 h. Mosquitoes were then counted and assessed for survival (alive or dead). Each trial was replicated eight times per species.

### 2.6. Statistical Analysis

A two-way analysis of variance (ANOVA) was used to evaluate the effects of sucrose concentration (10% vs. 40%), the presence of a heat source (hand warmer), and their interaction on mosquito feeding success. Feeding success across active ingredient treatments was analyzed using a one-way ANOVA, while mosquito survival at 24, 48, and 72 h post-exposure was evaluated using the log-rank test (Mantel–Cox). Additionally, the Wilcoxon matched-pairs signed-rank test was used to assess differences in feeding success between perforated bag and control groups in mosquitoes, butterflies, and honey bees. In the semi-field experiment, differences in mosquito mortality between the treatment and control traps were analyzed using the Wilcoxon matched-pairs signed-rank test for each species. Each trial replicate (*n* = 8) included a paired treatment and control trap. All statistical analyses were performed using JMP Statistics, Version 18.0 (SAS Institute Inc., Cary, NC, USA), and differences were considered statistically significant at *p* < 0.05.

## 3. Results

### 3.1. Mosquito Sugar Feeding Across Membrane Types, Sugar Concentrations, and Thermal Cues

In the comparison trials of different membranes for sugar feeding success, we observed that most membrane materials tested did not support successful feeding by *Cx. quinquefasciatus* when used to deliver a 10% sucrose solution (Table 1). Feeding success was 10% or less for cell culture bags, dialysis film, DuraSeal film, Parafilm, polyisoprene, and polyethylene. The addition of ATP to sucrose in Parafilm increased feeding success from 0% to 5% (Table 1). Lamb skin performed better than most other membranes, with a mosquito feeding success rate of 55%.

**Figure 1:**
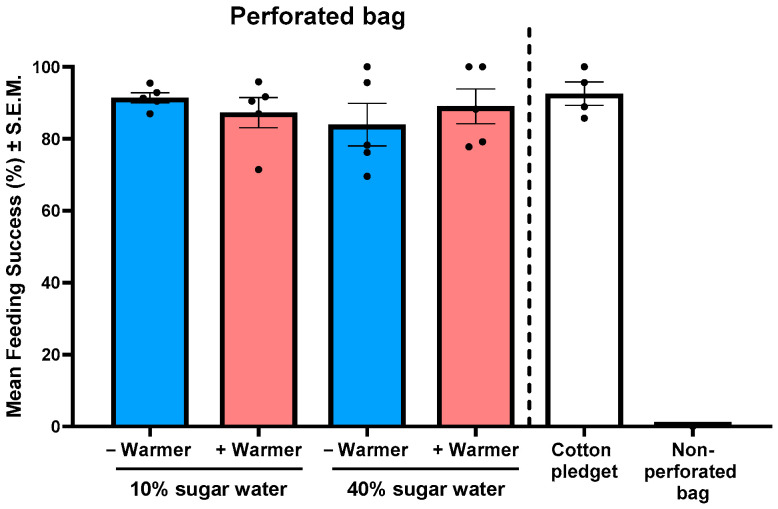
Mean feeding success of mosquitoes (*Cx. quinquefasciatus*) exposed to 10% or 40% sugar (sucrose) solutions delivered via a perforated bag, with (+Warmer) or without (−Warmer) hand warmers. A sucrose-saturated cotton pledget served as a positive control, and a non-perforated bag containing sucrose solution served as a negative control. Each dot represents the mean feeding success of a single replicate (*n* = 5).

In contrast, the perforated bag design enabled consistent and high feeding success, with 87% of mosquitoes successfully feeding; feeding success in the control (cotton pledget) was 95% (Table 1). Two-way ANOVA revealed no significant effects of sucrose concentration (10% vs. 40%), heat source (hand warmer), or their interaction on mosquito feeding success (sucrose: *F*_1,16_ = 0.4204, *p* = 0.5259; warmer: *F*_1,16_ = 0.0120, *p* = 0.9142; sucrose × warmer: *F*_1,16_ = 1.0999, *p* = 0.3099; Figure 1). The highest feeding success was observed with the 10% sucrose solution without a hand warmer (mean ± standard error of the mean (S.E.M.): 91.4% ± 1.4%). Post hoc Tukey’s HSD tests indicated that the presence of a hand warmer did not significantly affect feeding success at either 10% (*p* = 0.9548) or 40% (*p* = 0.9084) sucrose concentrations. Feeding success on the cotton pledget control was 92.6 ± 3.2%, whereas no mosquitoes fed from the non-perforated bag.

### 3.2. Acute Toxicity of ATSB Active Ingredients

**Figure 2:**
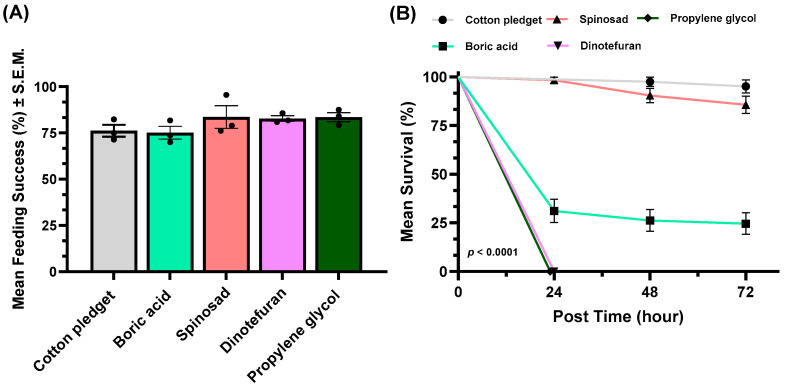
(**A**) Mean feeding success of mosquitoes (*Cx. quinquefasciatus*) exposed to active ingredients or control (cotton pledget) delivered via a perforated bag over a 72-h period. Each dot represents the mean feeding success of a single replicate (*n* = 3). (**B**) Survival of mosquitoes at 24, 48, and 72 h following exposure to each treatment and control. In no-choice assays, 20 female mosquitoes were provided access to a 10% sugar (sucrose) solution containing boric acid, spinosad, dinotefuran, or propylene glycol delivered via a perforated bag.

Mosquito feeding success was high (70.0–95.7%) for all active ingredients tested, and no difference was observed between toxic sugars and the sugar water control (Figure 2A). The highest mean feeding rate was observed in the spinosad treatment (83.6% ± 6.1%) and lowest in the boric acid treatment (75.2% ± 3.6%). There was no significant difference in feeding success among active ingredients mixed with a 10% sucrose solution in the perforated bag (one-way ANOVA: *F*_4,10_ = 1.3063, *p* = 0.3320; Figure 2A). Dinotefuran and propylene glycol were the most effective in reducing survival, inducing 100% mosquito mortality within 24 h (Figure 2B). Boric acid reduced mosquito survival by 68.4% after 24 h; however, no further significant decline was observed at 48 or 72 h compared with the 24-h mortality. In the spinosad treatment, mosquito survival was 98.4%, 90.4%, and 85.5% at 24, 48, and 72 h, respectively. The control group maintained 100%, 95%, and 95% survival at 24, 48, and 72 h, respectively.

### 3.3. Sugar Feeding Success and Mortality of Insect Pollinators on Perforated Bag-Based ATSB System

**Figure 3:**
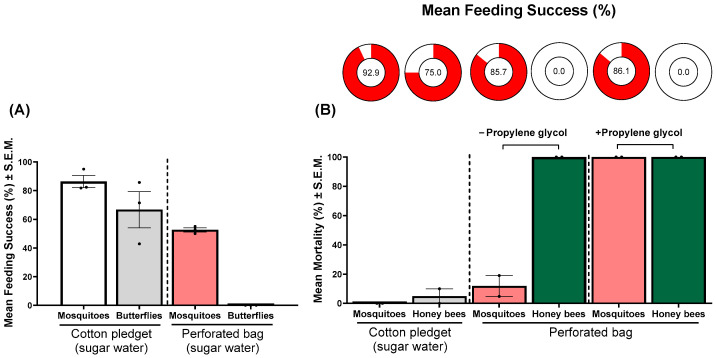
(**A**) Mean feeding success of mosquitoes (*Cx. quinquefasciatus*) and butterflies (mixed species) exposed to a 10% sugar (sucrose) solution delivered via a perforated bag. A sucrose-saturated cotton pledget served as a control. Each dot represents the mean feeding success of a single replicate (*n* = 3). (**B**) Mean feeding success (circles) and mortality of mosquitoes and honey bees (*Apis mellifera*) exposed to 10% sugar solutions. Treatments included a cotton pledget control, perforated bags without (−) propylene glycol, and perforated ATSB bags containing (+) 10% propylene glycol. Bars represent mean percentages ± S.E.M.

No butterflies were able to access or feed on sucrose solution when it was presented in a perforated bag (mean feeding success: 0.0% ± 0.0%; Figure 3A). In contrast, *Cx. quinquefasciatus* mosquitoes readily accessed the same perforated bag, with 52.6% ± 1.4% of individuals exhibiting successful feeding. When a cotton pledget saturated with 10% sucrose was used as a control, feeding success increased to 86.4% ± 4.3% for mosquitoes and 66.7% ± 12.6% for butterflies (Figure 3A).

Similarly, honey bees showed no evidence of feeding from either perforated bag, regardless of propylene glycol content (0.0% ± 0.0% in all cases), while *Cx. quinquefasciatus* readily fed from perforated bags, with or without propylene glycol (85.7% ± 4.8% and 86.1% ± 4.4%, respectively) (Figure 3B). Mean feeding success on cotton pledget was 92.9% ± 7.2% for *Cx. quinquefasciatus* and 75.0% ± 5.0% for honey bees. Mortality outcomes were consistent with observed feeding success. In *Cx. quinquefasciatus*, 100% mortality was observed in the groups exposed to perforated bag-based ATSB system containing 10% propylene glycol, whereas only 11.9% ± 7.1% mortality occurred in the bags without propylene glycol. No mortality was recorded in the control group (0.0% ± 0.0%). In contrast, honey bees exhibited 5.0% mortality in the control group and 100% mortality in both perforated bag treatments (with and without propylene glycol); this mortality was attributed to starvation resulting from an inability to access the sugar source, rather than toxicological effects (i.e., honey bees examined lacked food coloring in the abdomen) (Figure 3B).

### 3.4. Semi-Field Validation of Perforated Bag-Based ATSB System

**Figure 4:**
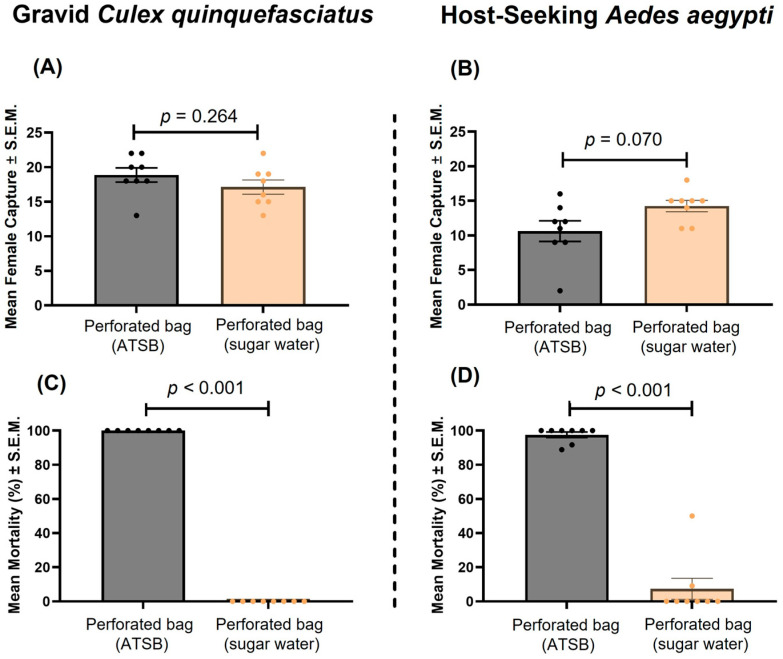
Mean number of female mosquitoes (*Cx. quinquefasciatus* and *Ae. aegypti*) captured and mortality (%) in BG-GAT passive traps deployed with perforated bag-based ATSB (with sugar water + propylene glycol) or control (sugar water only) under semi-field conditions. (**A**,**C**) Gravid *Cx. quinquefasciatus* exposed to BG-GAT traps baited with fermented grass infusion. (**B**,**D**) Host-seeking *Ae. aegypti* exposed to BG-GAT traps baited with a CO_2_-generating ExHale bag. Panels (**A**,**B**) show mean numbers of mosquitoes captured ± S.E.M., and panels (**C**,**D**) show mean mortality (%) ± S.E.M. For each replicate, 20 mosquitoes were released (*n* = 8 per treatment). Individual replicate values are overlaid on each bar.

Semi-field evaluations demonstrated that the perforated bag-based ATSB system induced significant mosquito mortality in both *Cx. quinquefasciatus* and *Ae. aegypti*, with minimal impact on trap capture rates. For gravid *Cx. quinquefasciatus* attracted to traps baited with fermented grass infusion, the mean number of captured mosquitoes did not differ significantly between ATSB and control treatments (mean ± S.E.M: 18.6 ± 1.1 and 17.8 ± 1.3, respectively; Wilcoxon matched-pairs signed-rank test, *p* = 0.264; Figure 4A). Mosquito mortality was 100% in all eight ATSB replicates, whereas no mortality (0%) occurred in traps with sugar water in perforated bags (*p* < 0.001; Figure 4C). Similarly, for host-seeking *Ae. aegypti* attracted to traps baited with a carbon dioxide–generating ExHale bag, the mean number of captured mosquitoes was not significantly different between ATSB and control treatments (mean ± S.E.M: 10.9 ± 1.7 and 14.1 ± 0.9, respectively; *p* = 0.070; Figure 4B). *Aedes aegypti* mortality was significantly higher (*p* < 0.001) in ATSB traps (92.5 ± 5.2%) compared to controls (1.4 ± 1.1%) (Figure 4D), with seven out of eight replicates achieving >90% mortality.

## 4. Discussion

Efficient sugar acquisition is a prerequisite for the success of ATSB strategies, and our results demonstrate that the perforated bag design facilitates sugar feeding in mosquitoes (Table 1 and Figure 1), whereas other tested membrane materials largely fail to do so (Table 1). Across a broad range of membranes tested including Parafilm, polyethylene, dialysis film, and latex, feeding success was minimal or absent, despite these materials being widely used to achieve high blood feeding success in previous studies [30,31] and despite the inclusion of phagostimulants such as ATP [32]. These findings reinforce previous observations that mosquito sugar feeding is strongly constrained by physical accessibility rather than chemical attractants alone. For example, large-scale ATSB field trials have used mosquito-bite–permeable bait stations that allow direct feeding, underscoring the importance of physical access to sugar [33]. Only lamb skin, a biologically derived and semi-permeable material [31,34], resulted in moderate feeding success. Unfortunately, the lamb skin membrane is expensive and requires delicate handling, so it is unlikely to be a scalable option for ATSB deployment. The perforated bag system consistently achieved high feeding success, irrespective of sucrose concentration, the presence of thermal cues, or toxicants. The absence of a difference between 10% and 40% sucrose solutions suggests that mosquitoes are not strongly selective for higher sugar concentrations once accessibility thresholds are met, consistent with prior studies indicating that sugar concentration primarily influences meal size rather than feeding initiation [35]. Similarly, the lack of heat associated increase in feeding indicates that, unlike blood feeding, sugar feeding in mosquitoes is not strongly driven by thermal host cues [36]. Collectively, these results indicate that mechanical accessibility, rather than chemical or thermal enhancement, is the dominant driver of sugar feeding success in this system.

While active ingredients in the perforated bag-based ATSB system did not deter sugar feeding (Figure 2A), survival outcomes varied markedly among active ingredients (Figure 2B), reflecting distinct differences in toxic potency and lethal kinetics [37]. Boric acid induced moderate mortality (68.4%) that plateaued after 24 h, consistent with its role as a slow acting stomach poison that disrupts gut integrity and osmotic balance [38]. Although boric acid is cost-effective and has a favorable mammalian safety profile [39], its incomplete and delayed lethality may limit effectiveness in high turnover mosquito populations. Similarly, spinosad exhibited the lowest acute toxicity in this study, with gradual mortality over 72 h, agreeing with previous findings that its efficacy is strongly dependent on dose, formulation, and repeated ingestion [17,21]. In contrast, dinotefuran and propylene glycol induced rapid and complete mortality within 24 h. While dinotefuran was highly effective, its classification as a neonicotinoid insecticide raises concerns regarding potential non-target exposure, particularly to pollinators and other beneficial insects [40]. Neonicotinoids are also characterized by varying degrees of environmental persistence, and sublethal exposure has been associated with adverse effects on honey bee foraging behavior, navigation, and colony level processes [41]. Although the perforated bag design substantially limits access compared with vegetation spray ATSB approaches, the potential for environmental leakage cannot be fully excluded under field conditions. Conversely, propylene glycol emerged as a particularly promising candidate for perforated bag-based ATSB systems. Classified as safe by the US Food and Drug Administration [42], propylene glycol induces mosquito mortality through osmotic stress and dehydration rather than neurotoxic mechanisms [43]. Our findings are consistent with previous work [43] demonstrating that ingestion of propylene glycol can drive rapid reductions in mosquito survivorship without deterring sugar feeding. The combination of rapid lethality, chemical stability, and a favorable environmental safety profile positions propylene glycol as a strong candidate for enclosed perforated bag-based ATSB systems designed to maximize mosquito control while minimizing ecological risks.

The perforated bag design restricted access to sugar solutions by non-target insect pollinators while remaining accessible to mosquitoes, as shown by the inability of butterflies and honey bees to feed from perforated bags (Figure 3A,B). This exclusion likely reflected differences in mouthpart morphology and feeding mechanics [44]: mosquitoes possess elongated proboscises with stylets typically on the order of ~10–50 µm in diameter [45], enabling penetration of small perforations and access to capillary-held fluids [46], whereas butterflies possess coiled proboscises typically hundreds of micrometers to millimeters [47] in diameter that are adapted for accessing open nectar [48], and honey bees rely on a lapping mechanism using a glossa approximately 100–200 µm in width [49] that requires exposed liquid surfaces [50]. We observed repeated contact and probing behavior by both butterflies and honey bees on the perforated bags, suggesting attraction to the sugar bait despite an inability to ingest the sugar. Accordingly, the small perforation size (<0.25 mm) and membrane tension selectively permitted mosquito feeding while excluding these non-target insects. Although honey bees experienced high mortality in no-choice assays, this was attributable to lack of access to sugar rather than toxicant exposure, as indicated by the absence of food coloring in the abdomen. Importantly, mortality rates were comparable between bees exposed to treatment (propylene glycol + sugar water) and those exposed to control (sugar water only), further supporting that mortality was driven by starvation rather than toxicant ingestion. However, these conditions likely overestimate such effects, as bees were confined without alternative food sources. Under field conditions, pollinators would be expected to forage elsewhere if access to the bait is restricted. While pollinators may still be attracted to the bait under field conditions, our results provide no evidence that they are able to feed on it. These findings should not be interpreted as evidence of complete ecological safety, and further field-based studies are needed to evaluate non-target effects under realistic conditions and in accordance with regulatory standards. Mosquito feeding success (52.6%; Figure 3A) in the perforated bag assay conducted in the presence of butterflies was lower than in earlier mosquito-only experiments (83%; Figure 1). This reduction is consistent with mosquitoes being co-confined with butterflies to approximate multi-taxon exposure. Butterfly movement (e.g., wing flapping) and repeated contact with the membrane likely increased disturbance to sugar-seeking mosquitoes [51], reducing their feeding success. Collectively, these findings indicate that the perforated bag-based ATSB design substantially reduced the risk of direct oral exposure to non-target insect pollinators while retaining mosquito accessibility.

Semi-field evaluations demonstrated that integrating the perforated bag-based ATSB system into passive traps resulted in high mosquito mortality without reducing capture efficiency. For both gravid *Cx. quinquefasciatus* and host-seeking *Ae. aegypti*, capture rates did not differ significantly between ATSB equipped and control traps, indicating that the presence of the ATSB did not interfere with behavioral attraction to oviposition or host cues (Figure 4A,B). Despite similar capture numbers, mortality in ATSB treatments was nearly complete for both species, with 100% mortality observed in all *Culex* replicates and >90% mortality in *Aedes* replicates (Figure 4C,D). These results highlight a key advantage of combining ATSB with passive trapping: mosquitoes are not merely removed from the population but are lethally exposed following capture, reducing the likelihood of escape from the passive trap. Importantly, this semi-field application demonstrates the potential to target gravid and host-seeking females, physiological states that are disproportionately important for population growth [52] and pathogen transmission [53], in contrast to conventional ATSB approaches that primarily target nectar-seeking mosquitoes. The high efficacy observed under semi-field conditions further suggests that perforated bag-based ATSB systems could be readily integrated into existing mosquito surveillance and control infrastructure, including gravid traps and CO2 baited stations. By localizing toxicant exposure within enclosed devices, this approach provides a scalable and environmentally responsible alternative with reduced susceptibility to weather-driven degradation and non-target impacts compared to broadcast ATSB spraying.

This study demonstrated that a perforated bag-based ATSB system achieved high mosquito feeding success, rapid mortality, and strong guild selectivity while minimizing access by non-target insect pollinators. By addressing key limitations of conventional ATSB approaches, including non-target exposure, environmental degradation, and inconsistent feeding, this platform represents a promising advancement in sugar-based mosquito control. Several limitations warrant consideration. First, laboratory feeding assays were conducted under controlled conditions in which mosquitoes were sugar-deprived prior to exposure, a design that maximized feeding success and may overestimate performance relative to natural environments where mosquitoes encounter competing sugar sources such as flowers and fermenting fruit, which may be more attractive due to associated olfactory cues and greater availability. In addition, environmental factors in the field (e.g., temperature, humidity, and light exposure) can influence mosquito behavior and bait attractiveness. Laboratory-reared mosquitoes may also differ from wild populations in nutritional status and responsiveness to artificial sugar sources. Consequently, perforated bag-based ATSB systems may not always outcompete natural sugar sources or achieve comparable efficacy under field conditions. Previous studies have shown that ATSB efficacy can vary across habitats, with reduced mosquito suppression in environments where ATSB compete with plant-derived sugars [54,55]. Second, toxicity and feeding assays were conducted primarily with *Cx. quinquefasciatus* and *Ae. aegypti*, and additional evaluations are needed to confirm efficacy across other epidemiologically important taxa, including *Aedes albopictus*. Third, non-target assessments were limited to butterflies and honey bees, and further studies are needed to evaluate potential impacts on other nectar feeding or predatory arthropods, including possible trophic transfer effects. Finally, semi-field trials were conducted under controlled conditions and may not fully capture the complexity of open field environments, including variable mosquito densities, variable vegetation densities, weather-driven degradation, and long-term bait durability. A preliminary field-simulated trial conducted under summer conditions in Florida showed that approximately 25% of bait volume was lost to evaporation over a three-week period, whereas the polyethylene membrane used in the perforated bag remained structurally intact, with no visible tearing, pore blockage, or leakage. Although the polyethylene does not fully block ultraviolet (UV) radiation, it can reduce UV-B transmission and may therefore provide partial protection against photodegradation of active ingredients [56]. Together, these observations suggest that the perforated bag design may support sustained field deployment over short durations; however, additional field studies are needed to fully assess durability and chemical stability under operational conditions. In addition, long-term storage stability was not evaluated in this study. While the intended deployment period is limited (≤3 weeks), accelerated shelf-life testing under elevated temperature conditions may be useful to assess bait efficacy, membrane integrity, and overall product quality during storage prior to field use. Importantly, this platform also offers clear opportunities for optimization. Incorporation of additional chemical lures (e.g., lactic acid) or sugar sources (e.g., natural fruit extract) could increase mosquito contact and feeding on the membrane surface, while scaling the volume and surface area of perforated bags may improve longevity and field practicality. Despite these limitations, the results presented here provide strong proof of concept supporting perforated bag-based ATSB systems as a targeted, effective, and ecologically responsible mosquito control tool. Further field scale evaluations will be essential to determine their operational value and integration into existing mosquito surveillance and control programs.

## Figures and Tables

**Table 1:** Feeding success of mosquitoes (*Cx. quinquefasciatus*) on various membranes. In no-choice assays, 20 or 23 female mosquitoes aged 3–7 days were exposed to a 10% sucrose solution dyed with blue food coloring, enclosed within each membrane. Mosquitoes were observed for 24 h, and the presence of blue food coloring on the abdomen was used as an indicator of feeding success, assessed under a dissection microscope.

Membrane Type	Fed (*n*)	Unfed (*n*)	Total (*n*)	Feeding Success (%)
Cell culture bag (FEP)	0	20	20	0
Dialysis film	2	18	20	10
DuraSeal film	0	20	20	0
Parafilm	0	20	20	0
Parafilm + ATP	1	19	20	5
Polyisoprene	0	20	20	0
Polyethylene	0	20	20	0
Lamb skin	11	9	20	55
Latex	0	20	20	0
Perforated bag	20	3	23	87
Control (cotton pledget)	19	1	20	95

## Data Availability

The original contributions presented in this study are included in the article/Supplementary Material. Further inquiries can be directed to the corresponding author.

## Supplementary Materials

The following supporting information can be downloaded at: https://www.mdpi.com/article/10.3390/insects17040370/s1, Figure S1: Perforated bag filled with 10% sugar (sucrose) solution mixed with blue food coloring to visually confirm ingestion. (B) *Culex quinquefasciatus* females visibly engorged with the dyed sugar solution. Assessment of feeding success in (C) butterflies and (D) honey bee; Figure S2: A BG-GAT trap equipped with a perforated bag-based ATSB system was deployed inside a 203.2 × 203.2 × 157.5 cm screen enclosure at the Florida Medical Entomology Laboratory (left). The perforated bag, containing a green-dyed toxic (propylene glycol) sugar solution, was affixed to the mesh platform inside the trap. Several dead mosquitoes (red circle) are visible on the trap bottom, indicating successful feeding and toxicant ingestion.

## Abbreviations

The following abbreviations are used in this manuscript:

ATSB	Attractive toxic sugar baits
BG-GAT	Biogents Gravid Aedes Trap
ANOVA	Analysis of Variance
