# Supplementary figures and images for: Evaluating a Novel Method to Limit Non-Target Mortality in Attractive Toxic Sugar Bait Systems
Source: Insects. 2026 Apr 1;17(4):370. doi: 10.3390/insects17040370 (PMC13115723; doi:10.3390/insects17040370)

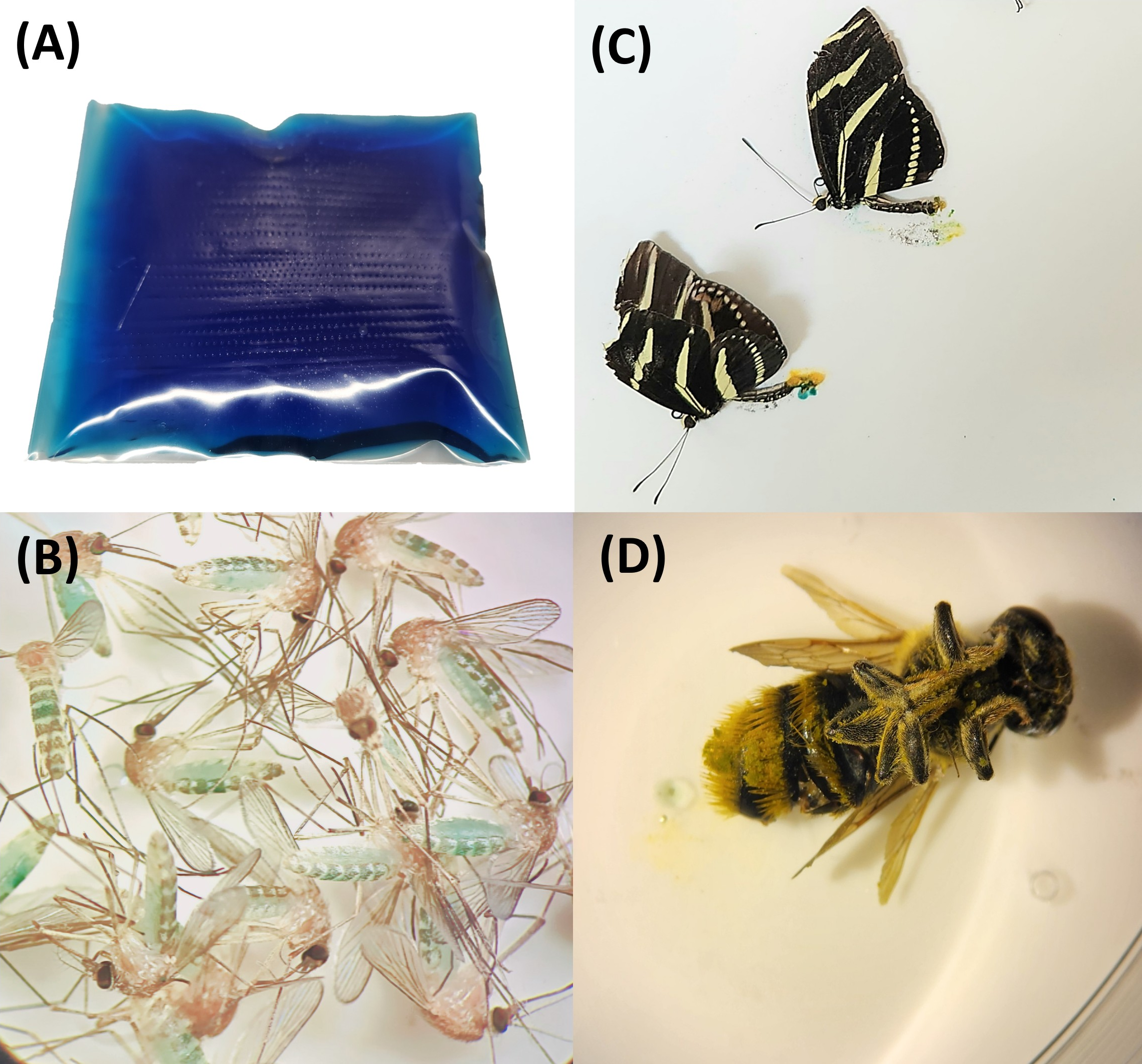

Supplement: Supplementary file 1 [file insects-17-00370-s001.zip › Supplementary Figures/(Supplementary) Figure S1.tif]

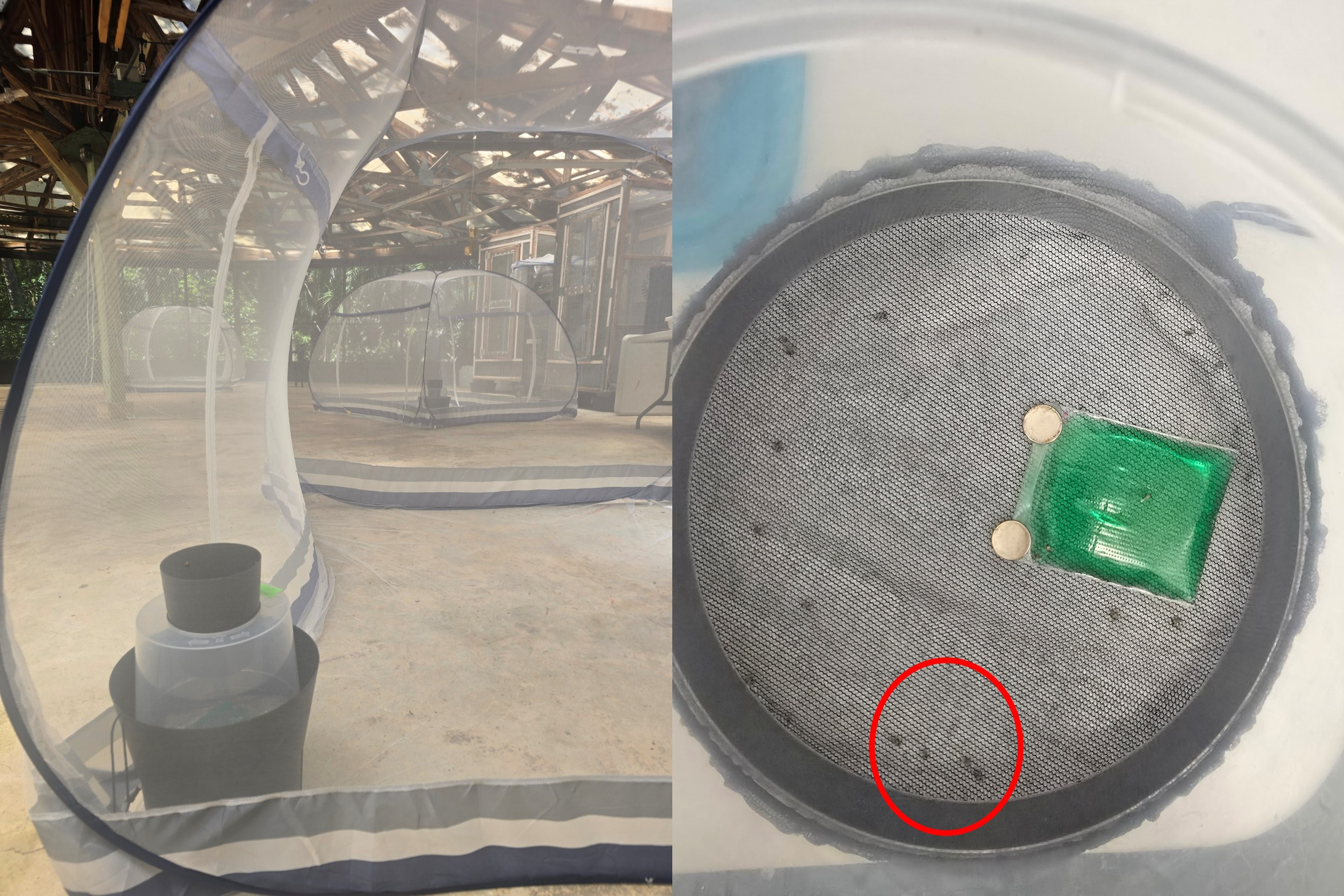

Supplement: Supplementary file 1 [file insects-17-00370-s001.zip › Supplementary Figures/(Supplementary) Figure S2.tif]
